# Epidemiology of rabies in camels

**DOI:** 10.5455/javar.2026.m1025

**Published:** 2026-03-16

**Authors:** Intisar Kamil Saeed, Yahia Hassan Ali, Tenzeil Awad Gadain Mohieddeen, Muaz Magzob Abdellatif, Baraa Abdel Aziz Ahmed Mohammed, Ali Mohammed AlHassan Eleragi, Husham Mohammed A. Ataalfadeel, Salma Yousif S. Elsheikh

**Affiliations:** 1 Department of Biological Sciences, College of Science, Northern Border University, Arar, Saudi Arabia; 2 Virology Department, Central Veterinary Research Laboratory, P.O. Box 8067, Khartoum, Sudan; 3 Department of Pathology, Central Veterinary Research Laboratory, P.O. Box 8067, Khartoum, Sudan; 4 Department of Mathematics, College of Science, Northern Border University, Arar, Saudi Arabia

**Keywords:** Rabies, camels, epidemiology

## Abstract

**Objectives:**

The present study aimed to investigate the epidemiology of rabies in camels by collecting and analyzing data on its occurrence and applying laboratory confirmation.

**Materials and Methods:**

During 2010–2022, data on the occurrence of rabies in camels, reported outbreaks, the number affected, seasonal occurrence, and outbreak management were collected and statistically analyzed. Data was collected from the Ministry of Animal Resources and Fisheries in Sudan.

**Results:**

During the study period, 11 outbreaks resulted in 24 clinically suspected cases of rabies in camels. The highest percentage (29.2%) was in 2021. Within localities, the highest occurrence rate (58.3%) was observed in Darfur State in Western Sudan. The majority of rabies outbreaks (62.5%) were reported during July–September. The logistic regression model revealed a 25.1% annual decline in rabies infection risk, with a notable increase in 2021, while increases of 137.4% linked to seasonal effects and 69% linked to geographical location were observed. Most of the affected camels (71%) died, the rest were destroyed, and vaccination covered the vast majority of in-contact animals.

**Conclusions:**

Rabies in camels was found to occur across different localities, with the highest figures in 2021 and peaks during July–September.

## 1. Introduction

The dromedary camel (*Camelus dromedarius*) is a species of significant economic impact; it is mainly raised in desert and semi-arid regions of Northern and Eastern Africa, Asia, and South America [1]. The estimated worldwide camel population was about 35 million; it was recently estimated at 40 million, with the majority in Africa and the Middle East [2]. Camels are used for milk, meat production, and riding [3]. Camels, like other livestock species, are known to be susceptible to infectious diseases with relative resistance to some infections [4].

Rabies is caused by a single-stranded, enveloped RNA virus classified in the Lyssavirus genus of the family *Rhabdoviridae* [5]. Rabies has been reported in camels in several African countries, including Ethiopia [6, 7], Tunisia [8], Niger [9], and Sudan [10, 11, 13]. A recent review highlighted camel rabies in Sudan, Mauritania, Morocco, Nigeria, and Niger [14]. The disease in camels has been reported in various Asian countries, including China [15, 16], Mongolia [17], India [18], Oman [19, 20, 21], Saudi Arabia [22], Jordan [10], Iran [23], and the Arabian Peninsula [24]. Khalafalla [14] reviewed camel rabies cases reported in Saudi Arabia, the UAE, Qatar, Oman, India, China, Iran, and Kazakhstan. The disease was also reported in camelids in South America [25].

In Sudan, the estimated camel population in 2019 was 4,895,000, most of which are kept in Western, Eastern, and Central Sudan [26]. The nomadic nature of most camel breeders and their continuous movement with their animals increased contact with other animal species, thereby spreading infectious diseases, including rabies. In this study, the epidemiology of rabies in camels in Sudan was investigated through the collection and analysis of data on reported rabies cases, as well as through laboratory testing of collected brain samples to identify the virus.

## 2. Materials and Methods

### 2.1. Ethical approval

This is an epidemiological study based on the collection and analysis of data on rabies occurrences. Brain samples were collected from camels that died of the disease and sent for routine laboratory diagnosis; no contact with live animals was adopted. The study was approved by the Local Committee of Bioethics (CVRL/2010/03) at the Veterinary Research Laboratory in Khartoum, Sudan.

### 2.2. Data collection

This work was conducted in the Rabies Unit, Central Veterinary Research Laboratory, Khartoum, Sudan. This unit is concerned with the laboratory diagnosis and research on rabies, including the collection of samples and data on rabies occurrence from the monthly and annual reports of the Animal Health Sector. Data concerning camel rabies outbreaks in Sudan between 2010 and 2022 were provided during January 2023 by the Animal Health Sector of the Ministry of Animal Resources and Fisheries. The details include the number of reported suspected rabid camels based on a history of animal bites preceding the obvious clinical signs, mostly nervous signs. Data also included year, season, state, vaccination, and the decisions made by owners and veterinary authorities.

### 2.3. Statistical analysis

The variables were noted, categorized, and documented in Microsoft Excel. It was opened with IBM’s SPSS^®^Version 27. Descriptive and analytical statistics were utilized to describe data.

### 2.4. Logistic regression analysis

Binary logistic regression was employed to identify the best-fitting model for estimating the association between suspected rabid camels and the variables under investigation.

### 2.5. Model coefficients

The Omnibus test was employed to evaluate the model’s fitness; the likelihood ratio, which follows a chi-square distribution, was used. The null hypothesis, that the model’s predictions precisely match observed group membership, was tested using the Hosmer-Lemeshow test. By contrasting the observed frequencies with those predicted by the linear model, a chi-square statistic was generated. *p* < 0.05 was the threshold for statistical significance, and the analyses had a 95% confidence level.

### 2.6. Laboratory diagnosis

#### 2.6.1. Collection of samples

Following standard precautions, brain tissues were collected aseptically from clinically suspected camels (*n* = 4) and transported on ice to the Rabies Unit at the Central Veterinary Research Laboratory, Khartoum, for virus identification. Unfortunately, most of the reported cases were in remote areas where it was not possible to collect and send samples for laboratory confirmation.

#### 2.6.2. Fluorescent antibody technique (FAT)

Brain samples (*n* = 4) were examined for rabies antigen detection using the fluorescent antibody test (FAT), as described previously [27].

### 2.7. RT-PCR

#### 2.7.1. Viral RNA extraction

Total RNA was extracted from brain tissues of clinically suspected camels and healthy, non-infected mice as a negative control. TRIzol kits were used according to the manufacturer’s instructions (Thermo Fisher Scientific Inc., 81 Wyman Street, Waltham, MA 02451).

#### 2.7.2. Synthesis of cDNA

Using the Transcriptor First Strand cDNA Synthesis Kit (Roche, Inc.) and the provided protocol, the cDNA was produced. Briefly, 5 µl of RNA was mixed with 1 µl random hexanucleotide primers (600 pmol/µl), 0.5 µl RNase inhibitor (40 U/µl), 4 µl 5x reaction buffer (8 mM MgCl₂), 5 µl reverse transcriptase (20 U/µl), 2 µl 10 Mm dNTP mix, and 7 µl nuclease-free water.

#### 2.7.3. cDNA Amplification

The amplification of cDNA (5 ul) was done as described by Heaton et al. [28]. In brief, the reaction mix (50 μl) was prepared as follows: PCR buffer containing 200 mM dNTP, 1.5 mM MgCl₂, 0.5 U of Taq polymerase (Invitrogen), and 2.5 pmol of each primer (JW10; GTC ATT AGA GTA TGG TGT TC and JW12; ATG TAA CAC CCC TAC AAT TG). Cycling was run as follows: heating at 95°C for 10 min, cycling for five times at 95°C for 90 sec, 45°C for 90 sec, 50°C for 20 sec, and 72°C for 90 sec, then 25 times at 95°C for 30 sec, 45°C for 60 sec, 50°C for 20 sec, and 72°C for 60 sec. A final cycle of 95°C for 30 sec, 45°C for 90 sec, and 50°C for 20 sec, with a final extension at 72°C for 10 min. Ethidium bromide-stained gel electrophoresis was performed to visualize the expected bands (~586 bp).

## 3. Results

### 3.1. Occurrence of camel rabies

**Figure 1.**
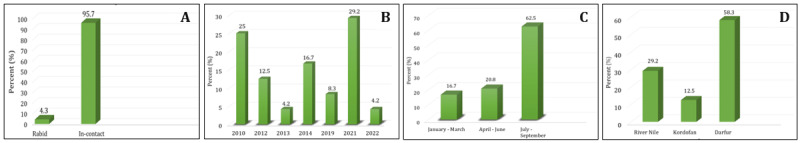
Camels showing signs of rabies (A), according to year (B), Season (C), and state (D).

**Figure 2.**
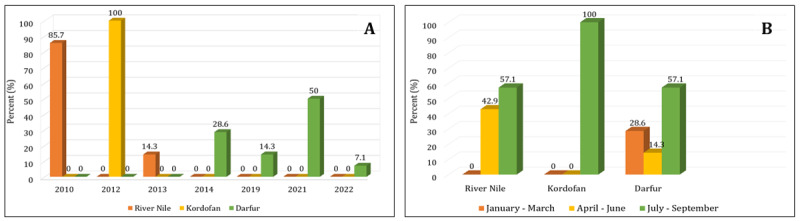
Rabies suspected camels according to state per year (A), and season (B).

A total of 24 suspected rabies cases in camels were reported in 11 outbreaks between 2010 and 2022. The highest occurrence rate (58.3%) was observed in Darfur State in Western Sudan. Over the study period, the highest overall occurrence rate (29.2%) was observed in 2021 (Tables 1, 2, 3 and Figures 1, 2).

**Table 1.** Cross-tabulations of camels showing signs of rabies according to year.

Year	2010	2012	2013	2014	2019	2021	2022	Total
Count	6	3	1	4	2	7	1	24
%	25.0	12.5	4.2	16.7	8.3	29.2	4.2	100

**Table 2.** Cross-tabulations of camels showing signs of rabies according to season.

Season	January – March	April – June	July – September	Total
Count	4	5	15	24
%	16.7	20.8	62.5	100

**Table 3.** Cross-tabulations of camels showing signs of rabies according to state.

State	River Nile	Kordofan	Darfur	Total
Count	7	3	14	24
%	29.2	12.5	58.3	100.0

### 3.2. Seasonal occurrence of rabies

Most of the overall rabies outbreaks (62.5%) during the study period were reported during July–September, then during April–June (20.8%); similar findings were noted in the three states (Tables 1, 2, 3 and Figures 1, 2).

### 3.3. Multivariate analysis

The *p*-value is 0.047, and the chi-square value is 24.252, indicating the significance of the model as confirmed by the Omnibus test. Hosmer-Lemeshow tests the null hypothesis that the model’s predictions match exactly the observed group memberships, comparing the observed frequencies with those predicted by the linear model.

#### 3.3.1. Logistic regression model

The analysis showed that we can fit the logistic regression model as:


\[
{\mathrm{ln}}({\mathrm{odds}}) = 574.720\,\, + \,\,0.525\,\, \times \,\,{\mathrm{state}}\,\, + \,\,0.865\,\, \times \,\,{\mathrm{season}} - 0.288\,\, \times \,\,{\mathrm{year}}
\]

Wald statistics indicate that season (*p*-value = 0.016), state (*p*-value = 0.009), and year (*p*-value = 0.003) are significant variables. Logistic regression analysis demonstrated a 25.1% annual reduction in the probability of rabies infection, despite an increase in 2021. The risk of contracting rabies rises by 137.4% by season and by 69% by state (Table 4).

**Table 4.** Statistical significance of variables included in the fitted model for the prevalence of camel rabies.

Variable	B	S.E.	Wald	*d_f_*	Sig.	Exp(B)	95% C.I. for EXP(B)	
							Lower	Upper
Year	–0.288	0.097	8.873	1	0.003	0.749	0.620	0.906
Season	0.865	0.359	5.793	1	0.016	2.374	1.174	4.801
State	0.525	0.201	6.805	1	0.009	1.690	1.139	2.507
Constant	574.720	194.007	8.776	1	0.003	3.960E + 249		

S.E., standard error; *d*_f_, degree of freedom; Sig., significance; C.I., confidence interval.

### 3.4. Management of rabies outbreaks

After exhibiting serious clinical signs of rabies, 70.8% of camels died naturally, while 29.2% of those that were still alive were condemned by their owners or veterinary authorities. Throughout the study period, 576 camels received rabies vaccinations, with the majority (436) administered in River Nile State. Contact animals received rabies vaccinations at varying coverage rates. All animals in contact with the disease were vaccinated from 2010 to 2014 in all reported outbreaks, except one in River Nile State. However, no camels received vaccinations between 2015 and 2022 (Table 5).

**Table 5.** Cross tabulations of destruction and vaccination taken by authorities.

Parameters		Died/ Destroyed		Vaccination	
		Died	Destroyed	Vaccinated	Not vaccinated
In-contact	Count	537	0	530	7
	%	100.0	0.0	98.7	1.3
Rabid	Count	17	7	0	24
	%	70.8	29.2	0	100.0
Total	Count	554	7	530	31
	%	98.8	1.2	94.5	5.5

### 3.5. Laboratory diagnosis

#### 3.5.1. Fat

All samples tested (*n* = 4) were positive for rabies virus antigen using FAT.

#### 3.5.2. RT-PCR

**Figure 3.**
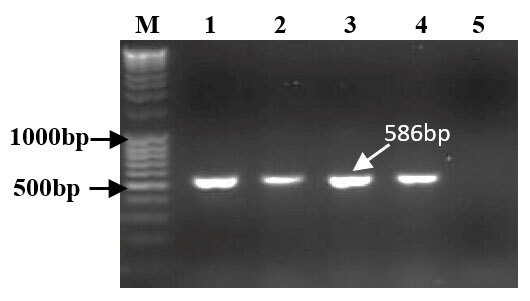
Ethidium bromide-stained agarose gel, Lane M: 100bp DNA ladder, Lane 1 to 4: camel brain samples, Lane 5: negative control. The amplicon is 586 bp.

All brain tissue examined (*n* = 4) produced amplicons of the expected size (~ 586 bp). Using negative control as a template, no amplification product was visualized (Figure 3).

## 4. Discussion

Rabies in camels has been reported in different countries, including China [16], Niger [9], and Mongolia [17, 29]. In the current investigation, between 2010 and 2022, 24 camels were reported as rabies suspects based on a history of animal bites and obvious rabies clinical signs in Sudan during 11 outbreaks. The Wald test indicated a significant association between the year and rabies infection, most likely linked to the control measures adopted. This is considered low compared to previously reported figures, where during 1996–2003, a total of 43 rabies-suspected camels were reported: 5 in Khartoum, 3 in Kordofan, 21 in River Nile, and 14 in Darfur [30]. In other reports, 79 cases were reported between 1992 and 2002 [11], 60 camel rabies suspects between 2003 and 2007 [12], and 11 cases between 2007 and 2010 [21, 31]. These considerably low reported cases most likely do not reflect the actual situation due to under-reporting, as noted in most published works in Sudan [32]. Within states, as noted in previous reports [11, 12, 30], most cases were reported in River Nile, Darfur, and Kordofan states, as expected. Darfur and Kordofan are the main areas of camel rearing, and the River Nile is a considerably smaller area where veterinary services are more accessible, facilitating the reporting of animal diseases. State was found to be a significant factor in the occurrence of rabies. The occurrence of camel rabies reported in this study is lower than previous reports in Sudan and Saudi Arabia, where, during 2010–2017, 34 cases (85%) were confirmed in camels [33]. However, our results are much higher than those reported in different countries. In Morocco, during 1951–2015, camel rabies cases accounted for only 0.3% of the total reported cases [34]. During 2012–2018, only 2 rabies cases were reported in Tunisia [8]. This could be attributed to the much higher camel population in Sudan. In Saudi Arabia, in Qasim, a questionnaire among 48 camel herdsmen with about 4000 animals found a camel rabies incidence of about 0.2% [22]. During 2005–2010, only 5 camel rabies cases have been recorded [35]. In Ethiopia, during 2018–2022, only 14 cases with 5 deaths were reported [6]. In Latin America, during 2009–2018, 11 rabies cases were reported in South American camelids [25].

The present study revealed a significant relationship between season and rabies outbreaks. Rabies in camels showed some seasonality; most cases were reported during July–September, which coincides with the association of rabies outbreaks with the mating seasons of dogs, which are usually reared with camel herds. The same observation was noticed recently in Sudan [32]. This observation was highlighted in different countries. In South Africa, the peak of rabies cases occurred during July-September [36]. A higher incidence of rabies cases was observed in spring and summer in China [16] and in Ethiopia [37]. Meanwhile, it was noticed to peak in spring and autumn in Morocco [34]. In Oman, during 2017–2019, rabies incidence peaked in April, with the lowest positivity in October [19].

In this study, only 4 camel brain samples were examined, all of which yielded positive results (100%). This is most likely due to the sending of brain samples from animals showing obvious rabies clinical signs. Most reported outbreaks were in remote areas, which explains the small number of samples tested. This underscores the need for either the availability of laboratory diagnosis in these areas or for improved tools for collecting and dispatching samples. Similarly, the high percentage of positivity noticed in this work was reported in Iran and China [15, 38]; however, in China also, camels accounted for 4% of laboratory-confirmed cases during 2010–2020 [16], and in Oman 49% of tested camel brains were rabies positive [21]; during 2006–2013, it was 60% [39]. However, higher positivity (86%) was reported more recently [19]. In Mongolia, 39% of tested camel brain samples were positive [17]. These variable results may be due to the stage of the disease and/or the variable sensitivity of the used techniques.

In response to rabies outbreaks in camels, it was noted that most camels (71%) that showed clinical signs died; the rest were destroyed by owners and/or veterinary authorities. The same management system was practiced in different countries; in Saudi Arabia, rabid camels were either isolated from the herd in the desert and left to die or destroyed [22].

Vaccination of in-contact camels was practiced in the vast majority of reported outbreaks during the study period, most of which were in River Nile State. During 2010–2014, in all reported outbreaks but one in River Nile State, vaccination coverage exceeded that of all in-contact animals. Between 2015 and 2022, this explains the spread of infection during this period. Administration of rabies vaccine to camels is not widely practiced; recently, trials to improve its vaccination are ongoing [40, 41].

## 5. Conclusions

Based on the results of this study, it was concluded that rabies is present in camels. The analysis revealed that year, season, and locality are significant determinants of rabies occurrence. Rabies occurrence was reported at a lower rate than previously reported; nonetheless, a comprehensive and detailed follow-up for the disease is needed.

## Data Availability

The data presented in this study are available from the corresponding author upon reasonable request.

## List of abbreviations

FAT, fluorescent antibody technique; RNA, ribonucleic acid; cDNA, complementary deoxyribonucleic acid; RT-PCR, reverse transcriptase polymerase chain reaction; bp, base pair; sec, seconds; μl, microliter; pmol/µl, picomoles per microliter; U/µl, units per microliter; min, minutes.

## Recommendations

Due to the under-reporting of animal diseases generally and rabies in particular, thorough investigations, including surveillance, epidemiology, and molecular studies, are highly recommended. Massive annual rabies vaccination programs for dogs, cats, and animals in contact in different areas of Sudan are highly recommended to control this serious disease.
